# Detection of RNA-DNA association by a proximity ligation-based method

**DOI:** 10.1038/srep27313

**Published:** 2016-06-03

**Authors:** Svetlana Petruk, Tyler K. Fenstermaker, Kathryn L. Black, Hugh W. Brock, Alexander Mazo

**Affiliations:** 1 Department of Biochemistry and Molecular Biology and Kimmel Cancer Center, Thomas Jefferson University, Philadelphia, PA 19107, USA; 2 Department of Zoology, University of British Columbia, 6270 University Boulevard, Vancouver, BC V6T 1Z4, Canada

## Abstract

We describe a proximity ligation assay (PLA)-based method of assessing association of DNA and RNA in single cells during the cell cycle. Pulse-labeling of DNA with EdU and RNA with BrU and testing their close proximity by PLA demonstrates that RNA synthesis in individual cells resumes about 30–45 min after DNA replication. Consistent with this conclusion, RNA Pol II phosphorylated at Ser2 of its CTD is detected at the same time as RNA transcripts on nascent DNA. Our results also show that RNA is associated with DNA foci during all stages of mitosis.

There are two main stages during the cell cycle where global transcription is susceptible to halting: DNA replication (S phase)^1^ and mitosis (M phase)^2^,^3^. These phases exhibit changes to chromatin architecture that may disrupt RNA transcription. *In vivo,* many proteins, including nucleosomes and components of the eukaryotic transcriptional machinery, are displaced from DNA during replication. Sites of active transcription may be marked by specific chromatin-associated proteins, or epigenetic marks, including TrxG and PcG epigenetic proteins^4^,^5^ that re-create the state of transcription in the next interphase. The functional outcome of epigenetic marking is initiation of transcription on nascent DNA, so it is important to know when transcription is resumed following DNA replication. This remains an open question, because there are currently no methods to determine the kinetics of RNA synthesis from newly synthesized DNA. A powerful EM technique^6^ detected transcripts within replicons, suggesting that replication fork passage may only transiently interfere with transcriptional activity^7^. This technique, however, cannot be used to examine the *in vivo* kinetics of the resumption of RNA synthesis following DNA replication. Additionally, some non-coding RNA molecules may play important epigenetic roles by recruiting transcriptional proteins to their sites of action after mitosis^8^, however, there are contradictory reports in the literature regarding the stability of RNA association with mitotic chromosomes^9^,^10^,^11^,^12^.

## Results

### The RNA-DNA Interaction Assay is an effective way to assess interactions between these molecules at a single cell level

**Figure 1:**
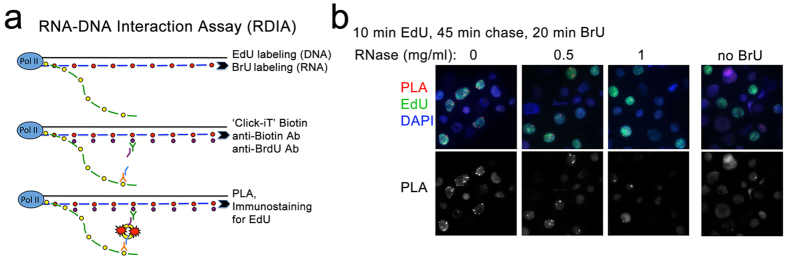
‘RNA-DNA Interaction Assay’ (RDIA). (**a**) A schematic representation of the RDIA technique. (**b**) RDIA was performed by labeling DNA with EdU, chasing 45 min and labeling RNA with BrU (left column). The specificity of RDIA was tested by omitting BrU (right column) and by treating cells with RNase A (middle columns). PLA signals are in red, biotin staining (EdU) is in green, DAPI is in blue; PLA signals only are shown in the bottom row.

To address these issues, we developed a new ‘RNA-DNA Interaction Assay’ (RDIA) that detects nascent RNA in close proximity to nascent DNA (scheme in Fig. 1a). In a typical experiment, DNA is labeled with EdU continuously or by pulse-chase, and RNA is then labeled with BrU. Cells are fixed, and biotin azide is covalently linked to an alkyne functional group on EdU in the presence of a copper catalyst via a “Click-it” reaction^13^. Nascent DNA is detected with anti-biotin antibody, and nascent RNA is detected with anti-BrdU antibody. The proximity ligation assay (PLA, Olink Bioscience) between these antibodies will indicate the presence of nascent RNA within 30–40 nm of nascent DNA *in vivo*. Additionally, cells can be immunostained for EdU, BrU, DAPI, and specific markers to provide specificity controls and to distinguish particular phases of the cell cycle. The high sensitivity and specificity of PLA uniquely permits the RDIA method to analyze the dynamics of RNA association with DNA at a single cell level in S and M phases.

The results of the RDIA assay in human GM22737 lymphoblast cells are shown in Fig. 1b. Cells were labeled with EdU for 10 min, chased for 45 min, and then RNA was labeled with BrU for 20 min. Following the ‘Click-it’ reaction and PLA, cells were immunostained for EdU, showing that PLA signals (red) occur only in the EdU labeled cells (green) (Fig. 1b, left column). Omission of BrU incorporation results in no PLA signals (Fig. 1b, right column), and digestion of RNA with RNase A results in loss of most of the PLA signals (Fig. 1b, middle columns), confirming that the RDIA is specific.

### Transcription resumes on individual genes approximately 30–45 minutes following DNA replication

**Figure 2:**
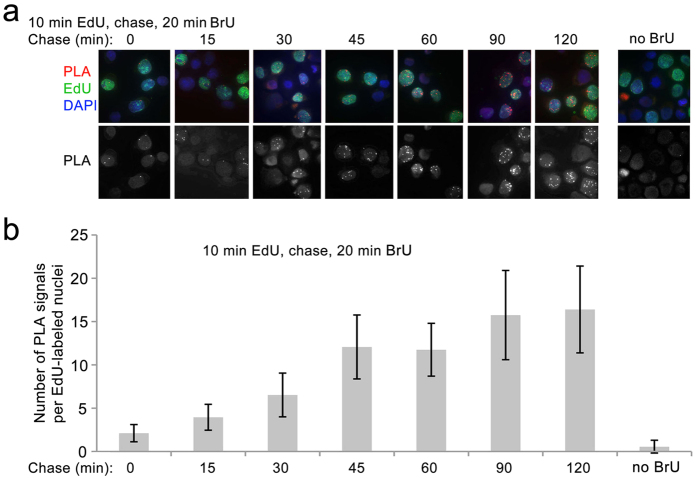
Measuring the kinetics of the resumption of RNA synthesis after DNA replication by RDIA. (**a**) The kinetics of the resumption of RNA synthesis after DNA replication. Labeling and chase times are indicted at the top. No BrU control is shown at the right. (**b**) The quantification of the results of the experiment in (**a**). The experiments were performed in three biological replicates. PLA signals were quantified in at least 50 EdU-labeled nuclei for each time point. Error bars represent standard deviation.

RDIA was used to examine when synthesis of RNA resumes after DNA replication. Nascent DNA was labeled for 10 minutes with EdU, followed immediately or after varying periods of chase by 20 min of RNA labeling with BrU. During 10 min of labeling with EdU, DNA is synthesized from a limited number of origins of replication^14^,^15^, so PLA signals in each individual cell reflects the resumption of RNA synthesis in different regions of the genome on relatively short stretches of nascent DNA. A small number of PLA signals compared to no BrU control was detected when DNA was labeled for 10 min with EdU with no chase followed immediately by 20 min labeling with BrU (Fig. 2a,b). An increased number of PLA signals was detected when EdU labeling was followed by 15 min chase, suggesting that RNA synthesis on nascent DNA begins 30–45 min after DNA replication. The number of PLA signals increases until 1.5 hr after DNA replication and remains steady thereafter (Fig. 2a,b). All cells have similar numbers of PLA signals after a given chase period, suggesting that initiation of RNA synthesis after replication begins and reaches its highest levels irrespective of the region of the genome being replicated.

**Figure 3:**
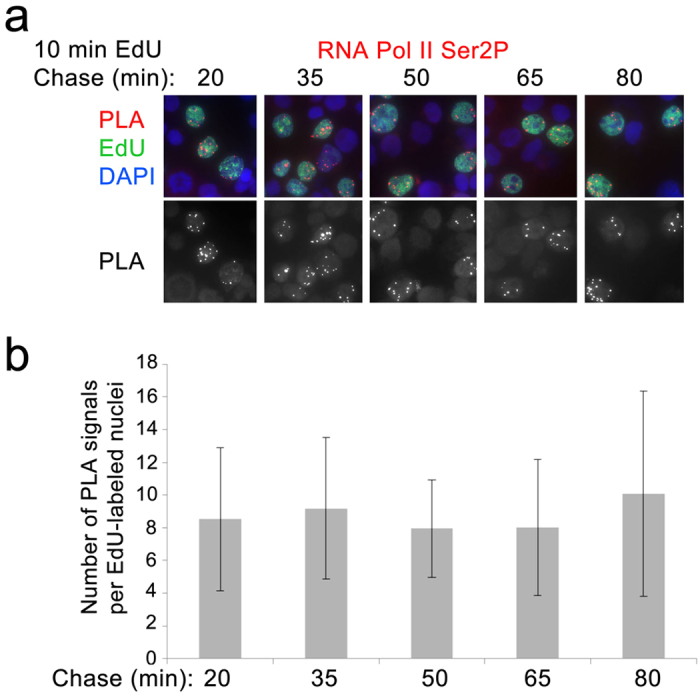
RNA Pol II phosphorylated at Ser2 is associated with nascent DNA. (**a**) Labeling and chase times are indicted at the top. Chromatin assembly assay (CAA) was performed between RNA Pol II Ser2P and nascent DNA (**b**) The quantification of the results of the experiment in (**a**). The experiments were performed in three biological replicates. PLA signals were quantified in at least 50 EdU-labeled nuclei for each time point. Error bars represent standard deviation.

To confirm that these results indeed reflect the resumption of transcription after DNA replication, we examined whether elongating RNA Polymerase II (RNA Pol II) associates with nascent DNA at the same time we begin to detect RNA (i.e. 30–45 min after replication). Our results show that the elongating form of RNA Pol II that is phosporylated at Ser2P of its CTD is associated with nascent DNA at 30 min after DNA replication (Fig. 3a,b). This is the same time that we detect an increase in association of newly synthesized RNA with nascent DNA, confirming that that synthesis of new RNA resumes shortly after DNA replication.

### RNAs stay stably associated with DNA through all stages of mitosis

**Figure 4:**
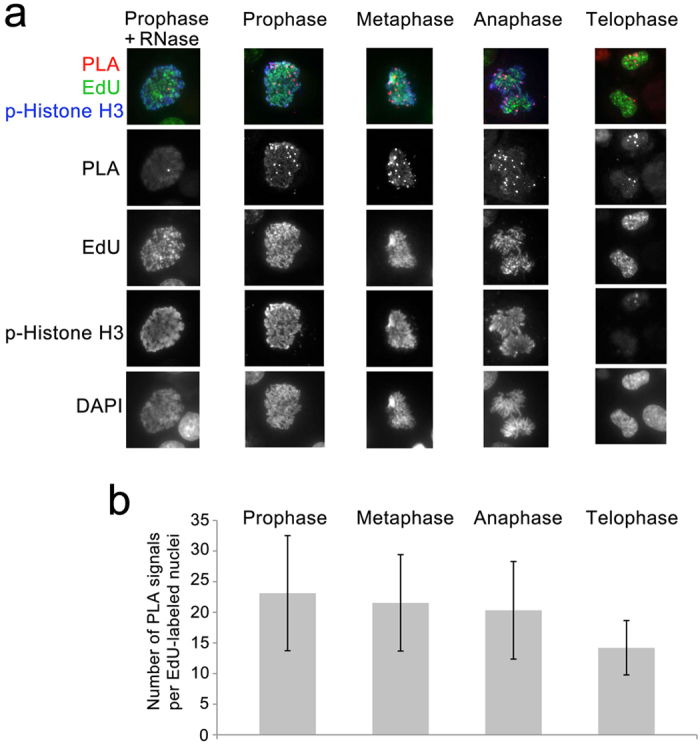
Using RDIA to examine the stability of RNAs during mitosis. (**a**) Following 15 min labeling with EdU, cells were grown for 5 hr and labeled with BrU. Following PLA (red), cells were immunostained for p-Histone H3 Ser10 (blue); EdU (biotin, green), and DAPI (grey in the bottom row). Specificity of the assay was tested by adding RNase A to a final concentration 1 mg/ml (prophase nuclei is shown in the left column). Split channels are shown below merged pictures. (**b**) The quantification of the results of the experiment in (**a**). The total number of PLA signals in both nuclei is presented for anaphase and telophase.

We extended the RDIA approach to examine whether RNAs remain associated with DNA during mitosis. DNA was labeled with EdU for 15 min and chased for 5 hr to ensure that cells with labeled DNA progressed to mitosis. RNA was then labeled for 55 min to ensure that labeled RNA can be detected through all stages of mitosis (approximately 1 hr long). Following PLA, cells were immunostained for EdU, the mitotic marker histone H3 phosphorylated at Ser10 and DAPI (EdU in green, p-Histone H3 in blue, and DAPI in grey in Fig. 4a). These markers allow us to unambiguously distinguish all stages of mitosis. Treatment with RNase A completely abrogated PLA signals providing confirmation of the specificity of these assays (Fig. 4a). RNA is detected in multiple foci on DNA through all stages of mitosis. The number of PLA signals diminishes during later stages of mitosis, especially during telophase (Fig. 4). This may be explained either by dissociation of some RNAs during late stages of mitosis or by higher compaction of chromatin which may result in merging of different foci on mitotic DNA.

## Discussion

Our finding that RNA synthesis resumes about 30–45 min after DNA replication is important because it addresses a long-standing dispute of whether components of transcriptional machinery are dissociated from DNA during replication. The existing data, which was derived mostly from studies in bacteria, are contradictory. Some *in vitro* studies suggest that RNA polymerase can withstand the passage of the replication fork^16^,^17^,^18^, whereas similar *in vitro* studies indicate that it is dissociated from DNA^19^,^20^. RNA polymerase stability during replication in eukaryotes may differ from prokaryotes because of the existence of chromatin packaging. Additionally, in eukaryotes transcription and replication may be compartmentalized to specific subnuclear foci or transcription and replication ‘factories’^21^,^22^, and thus may be topologically separated, preventing collision of replication and transcription complexes.

It was shown that 30 min labeling of the 3Y1B rat embryonic fibroblasts with BrdU results in more than 100 replication foci^14^. It is estimated that each such focus in the mammalian cells may contain up to 5 active replicons^15^. In our experiments, human GM22737 cells were labeled with EdU for 10 min (Figs 1 and 2), probably representing a smaller number of replicons that are activated during this short period of the 8–10 hr long S-phase. Nevertheless, we detected a significantly smaller number of PLA signals (10–20) marking transcriptional foci on nascent DNA, than the number of estimated replicons. Given very high sensitivity of PLA, and the fact that in other types of experiments we detected very large number of PLA signals, these discrepancies are unlikely to be explained by the technical limitations of our assay. We suggest that a smaller number of transcriptional foci on nascent DNA compared to an expected number of replicons is likely explained by translocation of multiple genes from the replication foci (or ‘factories’) to transcription ‘factories’. This explanation is further supported by the observation that the number of foci on nascent DNA detected with nascent RNA is very similar to the number of foci detected with RNA Pol II Ser2P (Fig. 3). Our data suggest that despite the potential topological separation of transcription and replication foci, there is a period when high levels of RNA synthesis are halted after replication. Further experiments are required to understand the molecular basis for this delay in resumption of transcription after replication.

Early studies focusing on transcription during mitosis showed that incorporation of radiolabeled ribonucleic precursors into RNA ceased on mitotic chromosomes^23^. Recent studies, however, do not support the idea that mitotic chromosomes are transcriptionally silent. Chan *et al*.^24^ synchronized cells using colcemid and allowed cells to incorporate FITC-rUTP and saw staining at kinetochores, but this result was lost upon α-amanitin treatment to inhibit transcription. Additionally, transcription during early mitosis is necessary to later induce full transcriptional inhibition in mitosis^25^. Studies of RNAs in mitosis are infrequent, and it is not clear if previously synthesized RNAs remain stable during mitosis. Early studies utilizing fluorescence *in situ* hybridization (FISH) showed that *Ubx* transcripts in *Drosophila* embryos were aborted during mitosis^26^. Additionally, while some studies fail to detect the *Xist* transcripts on mitotic chromosomes^9^,^11^,^12^, another study using different conditions for FISH detected *Xist* on X chromosomes at all mitotic stages in mouse embryonic stem cells^10^.

Given the contradictions in the current literature on RNA transcription in S and M phases, the RDIA technique has significant technical advantages of reproducibility, unimolecular sensitivity, and the ability to analyze single cells. Apart from being the only existing method to examine the resumption of RNA synthesis after DNA replication, RNA synthesis on nascent DNA can be examined in single cells with unimolecular sensitivity. This technique surpasses standard IF in both its sensitivity and its ability to analyze the proximity of the two molecules. While colocalization by IF is often interpreted as physical interaction and/or proximity, this assumption ignores the possibility that the molecules in question are actually separated in the third dimension, a possibility which is undetectable by conventional microscopy. Thus, this technique will be useful in addressing biological questions involving the assessment of not only the close association of RNA to DNA, but also the examining this in a kinetic manner. Additionally, immunohistochemical detection of desired markers (not shown) allows identification of specific cell types to address questions about RNA synthesis during development and cell differentiation.

## Methods

### Cell culture and DNA and RNA labeling

Human GM22737 (EBV transformed B lymphocyte) cells were cultured in RPMI 1640 medium with L-glutamine (Cellgro) supplemented with 10% fetal calf serum (Gemini) at 37 °C in a humidified incubator containing 5% CO_2_. Cells were passaged 16 h before the experiment; all experiments were performed on exponentially growing cells. Approximately 30,000 cells per each condition were used. To label nascent DNA 5 μM EdU (5-ethynyl-2′-deoxyuridine; Invitrogen) was added to the culture medium for 10 min. Cells were spun down for 3 min at 1000 rpm, washed with fresh medium, and chased for different times. To label RNA BrU (5′-Bromouridine, 250 mM stock in 1x PBS; TCI America) was added to a final concentration of 5 mM and incubated for 20 min at 37 °C. After incubation cells were immobilized on SuperFrost Plus Microscope Slides (Fisher Scientific) by cytospin centrifugation at 1000 rpm for 4 min. Cells were fixed in 4% formaldehyde for 12 min, washed in PBS, permeabilized in 0.25% Triton X100 (Fisher Scientific) for 10 min, and washed in PBS. For “no BrU” control, cells were labeled with 5 μM EdU for 10 min, spun down at 1000 rpm for 3 min, washed with fresh medium, chased for 1 h at 37 °C, and immobilized on slides by cytospin centrifugation. The “no BrU” control was further processed as all other slides.

### Labeling of mitotic chromosomes

To label mitotic chromosomes, cells were incubated with 5 μM EdU for 15 min, spun down for 3 min at 1000 rpm, resuspended in fresh medium, and chased for 5 hours at 37 °C at 5% CO_2_. Cells were then labeled with 5 mM BrU for 20 min, spun down for 3 min at 1000 rpm, and fresh medium was added with 5 mM BrU and incubated for an additional 30 min at 37 °C to ensure enough BrU was present in medium for the duration of mitosis. Cells were then immobilized on slides by cytospin centrifugation, fixed, and permeabilized as above.

### RNase experiments

For the RNase experiments, cells were labeled with 5 μM EdU for 10 min, washed with fresh medium, chased for 45 min, incubated with BrU for 20 min, spun down for 3 min at 1000 rpm, and permeabilized in PBS containing 0.3% Tween 20 for 5 min. Cells were spun down again and then treated with either PBS with 3 mM MgCl_2_ (control), 0.5 mg/ml or 1mg/ml RNase A (DNase free, Roche) for 20 min at room temperature. After incubation cells were immobilized on slides by cytospin centrifugation. Cells were subsequently fixed and permeabilized as above. Mitotic RNase A experiments were performed after EdU labeling and 5 hr chase as described above and incubation with 5 mM BrU for 20 min.

### Click Reaction

In preparation for the click reaction, samples were briefly blocked with 1% bovine serum albumin (BSA; Fisher Scientific) in PBS. To covalently link biotin to EdU a click reaction was perfomed in 1x PBS by adding in the following order: copper (II) sulfate (final concentration 2 mM; Acros Organics), biotin azide (final concentration 5 μM; Invitrogen), and freshly prepared L-ascorbic acid sodium salt (final concentration 10 mM; Acros Organics) for 25 minutes at room temperature. Slides were then washed with PBS, and blocked in 1x Western blocking reagent (Roche) in PBS containing 1.5% donkey serum and 0.01% Tween 20 for 30 min at room temperature. Primary antibodies for RDIA, mouse anti-BrdU (MoBu1 1:150; ThermoScientific) to detect BrU and rabbit anti-biotin (1:1000; Abcam) to detect EdU, were diluted in 1x Western blocking reagent in PBS containing 0.01% Tween 20, and incubated overnight at 4 °C.

### Chromatin Assembly Assay (CAA) with RNA Pol II Ser 2P

CAA was performed as described previously^4^,^5^. Approximately 30,000 human GM22737 cells per condition were labeled by adding 10 μM EdU to culture medium for 10 minutes and incubated at 37 °C. Cells were spun down for 3 minutes at 1000 rpm, washed with fresh medium, and chased for different times. Cells were immobilized on slides, fixed, permeabilized (as described above), and then processed for the Click reaction followed by 4 °C overnight incubation with mouse anti-biotin (1:1000; Jackson Immunoresearch) and rabbit anti-RNA Pol II Ser2P (1:750; Bethyl Laboratories) in 1x Western blocking reagent diluted in PBS.

### Proximity Ligation Assay (PLA) and Immunostaining

Following incubation with primary antibodies, slides were washed in PBS for 20 minutes at room temperature, and spots with cells were circumscribed by ImmEdge pen (Vector Laboratories) to keep reagents localized on cells. The proximity ligation assay (PLA; Olink) was performed using 20 μL of reaction per step per slide according to manufacturer’s instruction. In brief, two PLA secondary probes, anti-mouse MINUS and anti-rabbit PLUS, were diluted 1:5 in 1x Western blocking reagent in PBS, and incubated for 1 hour at 37 °C. Slides were washed in PBS for 10 minutes, followed by the ligation reaction, in which PLA ligation stock was diluted 1:5 in dH_2_O and 1:40 ligase was added and incubated for 30 min at 37 °C. Slides were washed in PBS for 5 minutes, followed by addition of the PLA amplification reaction (1:5 amplification stock and 1:80 polymerase in dH_2_O) for 100 min at 37 °C. Slides were washed with PBS for 5 minutes. In order to detect EdU, anti-rabbit Alexa Fluor 488 (1:1000; Jackson Immunoresearch) or anti-mouse Alexa Flour 488 (1:1000; Jackson Immunoresearch) for RDIA and CAA, respectively, was diluted in 1x Western blocking reagent in PBS and added to the samples and incubated for 1 hour at room temperature. To ensure visualization of EdU following PLA, slides were further incubated with mouse anti-biotin Alexa Fluor 488 (1:1000; Jackson Immunoresearch) diluted in 1x Western blocking reagent in PBS for 30 minutes. Slides were washed for 20 minutes in PBS and mounted in Vectashield mounting medium containing DAPI (Vector Laboratories). In mitotic experiments, EdU was detected using anti-rabbit Alexa Fluor 647 (1:1000; Jackson Immunoresearch), and mitotic chromosomes were visualized using anti-phospho-Histone H3(Ser10) clone3H10 FITC-conjugated (1:1000; Millipore). An Olympus microscope equipped with a digital camera was used to obtain images of cells. PLA signals were counted in 50–100 EdU-labeled nuclei from three independent experiments and standard deviation calculated.

## Additional Information

**How to cite this article**: Petruk, S. *et al*. Detection of RNA-DNA association by a proximity ligation-based method. *Sci. Rep.* **6**, 27313; doi: 10.1038/srep27313 (2016).
